# Arteriovenous malformation (hemangioma) of the radial artery: case report and treatment outcome

**DOI:** 10.1093/jscr/rjag936

**Published:** 2026-10-27

**Authors:** Teodora Todorova, Tamara Angelovska, Slavica Kostadinova-Kunovska, Nikola Gramatnikovski, Dejan Daskalov, Marta Foteva, Vilijam Velkovski, Nenad Atanasov, Alan Andonovski, Milan Samardziski

**Affiliations:** University Clinic for Orthopedic Surgery, Mother Teresa No. 17, Skopje 1000, North Macedonia; Faculty of Medicine, Institute of Pathology, University Ss. Cyril and Methodius in Skopje, 50 Divizija No. 6, Skopje 1000, North Macedonia; Faculty of Medicine, Institute of Pathology, University Ss. Cyril and Methodius in Skopje, 50 Divizija No. 6, Skopje 1000, North Macedonia; University Clinic for Thoracic and Vascular Surgery, Skopje 1000, North Macedonia; Institute of Radiology, Faculty of Medicine, Ss. Cyril and Methodius University in Skopje, North Macedonia; University Clinic for Orthopedic Surgery, Mother Teresa No. 17, Skopje 1000, North Macedonia; University Clinic for Orthopedic Surgery, Mother Teresa No. 17, Skopje 1000, North Macedonia; University Clinic for Orthopedic Surgery, Mother Teresa No. 17, Skopje 1000, North Macedonia; University Clinic for Orthopedic Surgery, Mother Teresa No. 17, Skopje 1000, North Macedonia; University Clinic for Orthopedic Surgery, Mother Teresa No. 17, Skopje 1000, North Macedonia

**Keywords:** arteriovenous malformation, hemangioma, sclerotherapy

## Abstract

Arteriovenous malformation hemangioma (AVMH) is a rare benign vascular lesion typically diagnosed in young adults, with 80%–90% of patients presenting before the age of 30. It most commonly affects the extremities, followed by the head and neck region. Establishing the differential diagnosis of AVMH can be challenging because it must be distinguished from other soft tissue lesions of the extremities. We present the case of a 44-year-old woman with recurrent AVMH, successfully treated with foam sclerotherapy using 10 ml of 3% polidocanol (Ethoxysclerol), prepared according to the Tessari method, followed by ligation of the radial artery. This report suggests that this combined therapeutic approach may represent an effective treatment option for selected patients with recurrent or extensive lesions when complete surgical excision is difficult or associated with significant morbidity.

## Introduction

Vascular lesions pose significant diagnostic and therapeutic challenges [1]. The nomenclature of vascular anomalies has long been inconsistent in the literature. While some authors have used the term hemangioma broadly to describe various vascular malformations, others have continued to employ outdated terms such as cavernous hemangioma for venous malformations and port-wine stain for capillary and arteriovenous malformations, contributing to persistent nosological confusion [2, 3]. Establishing the differential diagnosis of arteriovenous malformation hemangioma (AVMH) can be challenging because it must be distinguished from other soft tissue lesions of the extremities, particularly schwannomas [4].

We present the case of a 44-year-old woman with recurrent AVMH, who was successfully treated with foam sclerotherapy using 10 ml of 3% polidocanol (Ethoxysclerol), prepared according to the Tessari method, followed by ligation of the radial artery.

## Case report

We present a case of a 44-year-old female patient who was referred to our outpatient clinic due to pain, swelling of the left forearm, and paraesthesia involving the fourth and fifth digits of the left hand. The patient reported a history of previous surgical excision of a lesion in the same region 10 years earlier. Histopathological examination at that time was consistent with an AVMH, excised with negative surgical margins. On physical examination, two well-healed surgical scars, each measuring ~10 cm, were observed on the left forearm, corresponding to the previous tumor excision. Based on the clinical presentation, a local recurrence was suspected. Contrast-enhanced magnetic resonance imaging (MRI) demonstrated an elongated inter- and intramuscular soft tissue lesion located anterolaterally at the level of the radial diaphysis. The lesion exhibited heterogeneous signal intensity and a lace-like appearance, consisting of tortuous serpiginous dilated vascular structures, most consistent with venous channels. Some of these vessels demonstrated MRI features of focal intraluminal thrombosis, while others contained small intraluminal calcifications (phleboliths). The lesion extended partially within the inter-muscular plane and partially infiltrated the muscle belly of the flexor pollicis longus anterior to the radius. Posteriorly, it also involved the abductor pollicis longus muscle and extended into the subcutaneous adipose tissue of the anterolateral aspect of the mid-forearm. The lesion measured ~14.5 cm in craniocaudal length, 3.5 cm in transverse diameter, and 4.5 cm in anteroposterior diameter. Following intravenous contrast administration, the lesion demonstrated heterogeneous enhancement. The MRI findings were consistent with a local recurrence of the previously excised vascular lesion (Figs 1–3).

**Figure 1:**
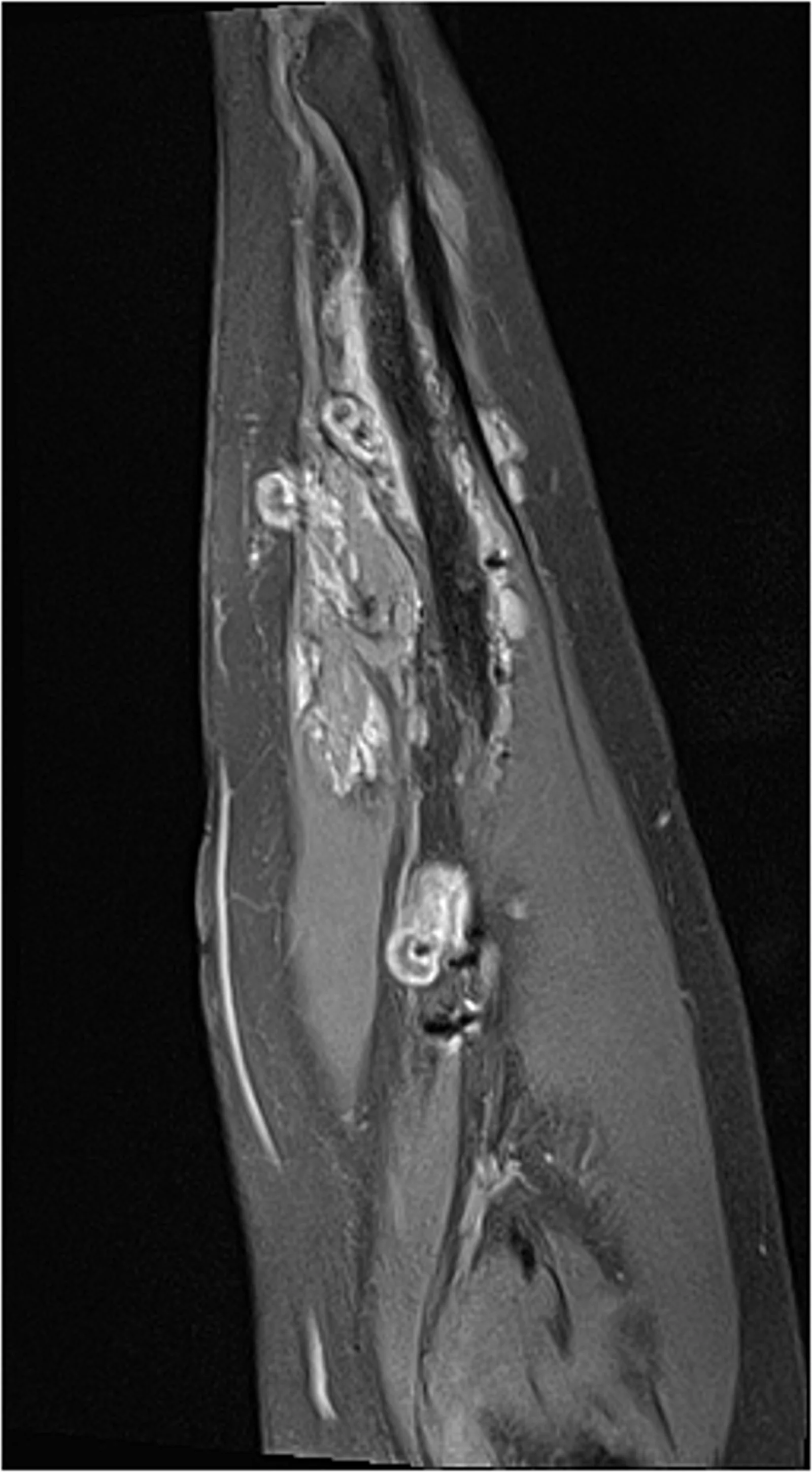
Preoperative sagittal magnetic resonance image of the left forearm demonstrating an extensive arteriovenous malformation (hemangioma) with a heterogeneous, infiltrative appearance and multiple serpiginous vascular structures extending through the soft tissues of the forearm.

**Figure 2:**
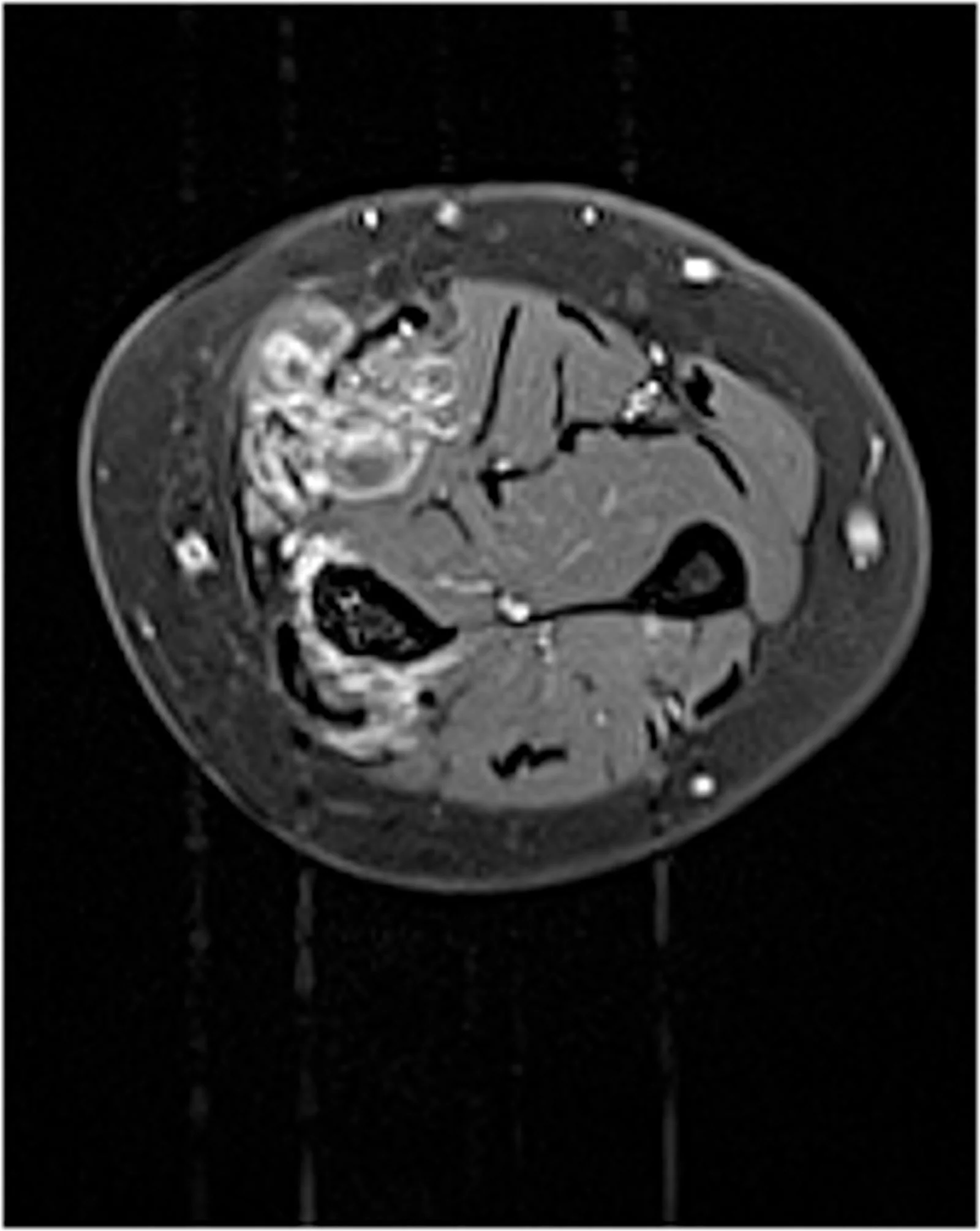
Preoperative axial magnetic resonance image of the left forearm demonstrating an arteriovenous malformation characterized by multiple tortuous vascular channels within the soft tissues, with involvement of the adjacent muscular compartments.

**Figure 3:**
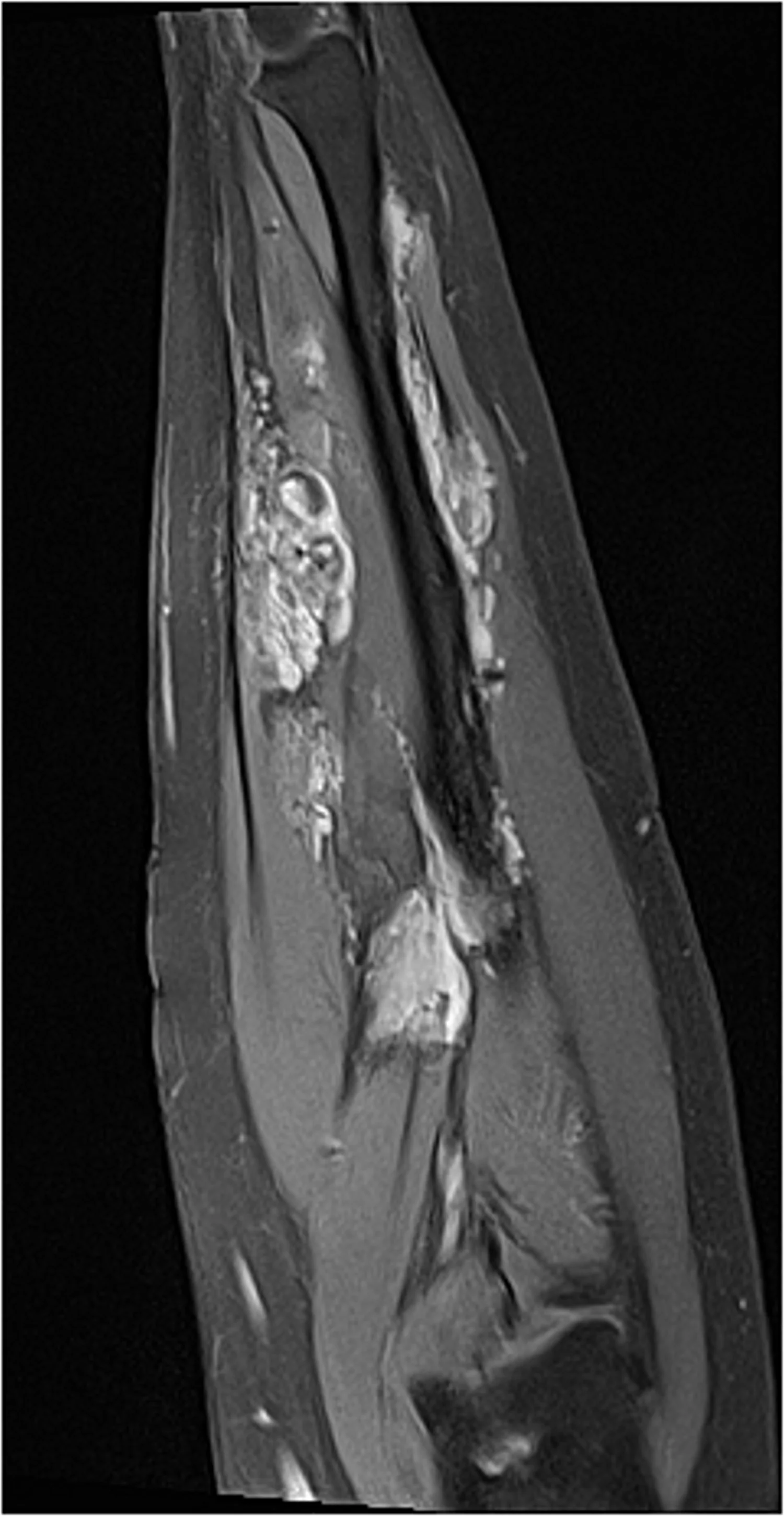
Preoperative magnetic resonance image of the left forearm demonstrating an extensive arteriovenous malformation with multiple dilated and tortuous vascular channels extending longitudinally through the soft tissues and involving the adjacent muscular compartments.

Following a multidisciplinary tumor board discussion at our referral center and detailed preoperative evaluation, surgery was performed. Intraoperatively, 3% polidocanol (Ethoxysclerol) 10 ml foam prepared according to the Tessari method sclerotherapy was administered, followed by ligation of the radial artery and neurolysis of the radial nerve. Histological analysis of the biopsy specimen confirmed the presence of an AVMH.

The early and late postoperative course was uneventful, with no perioperative complications. At the 3-month follow-up, contrast-enhanced MRI of the left forearm demonstrated complete regression of the lesion (Figs 4 and 5).

**Figure 4:**
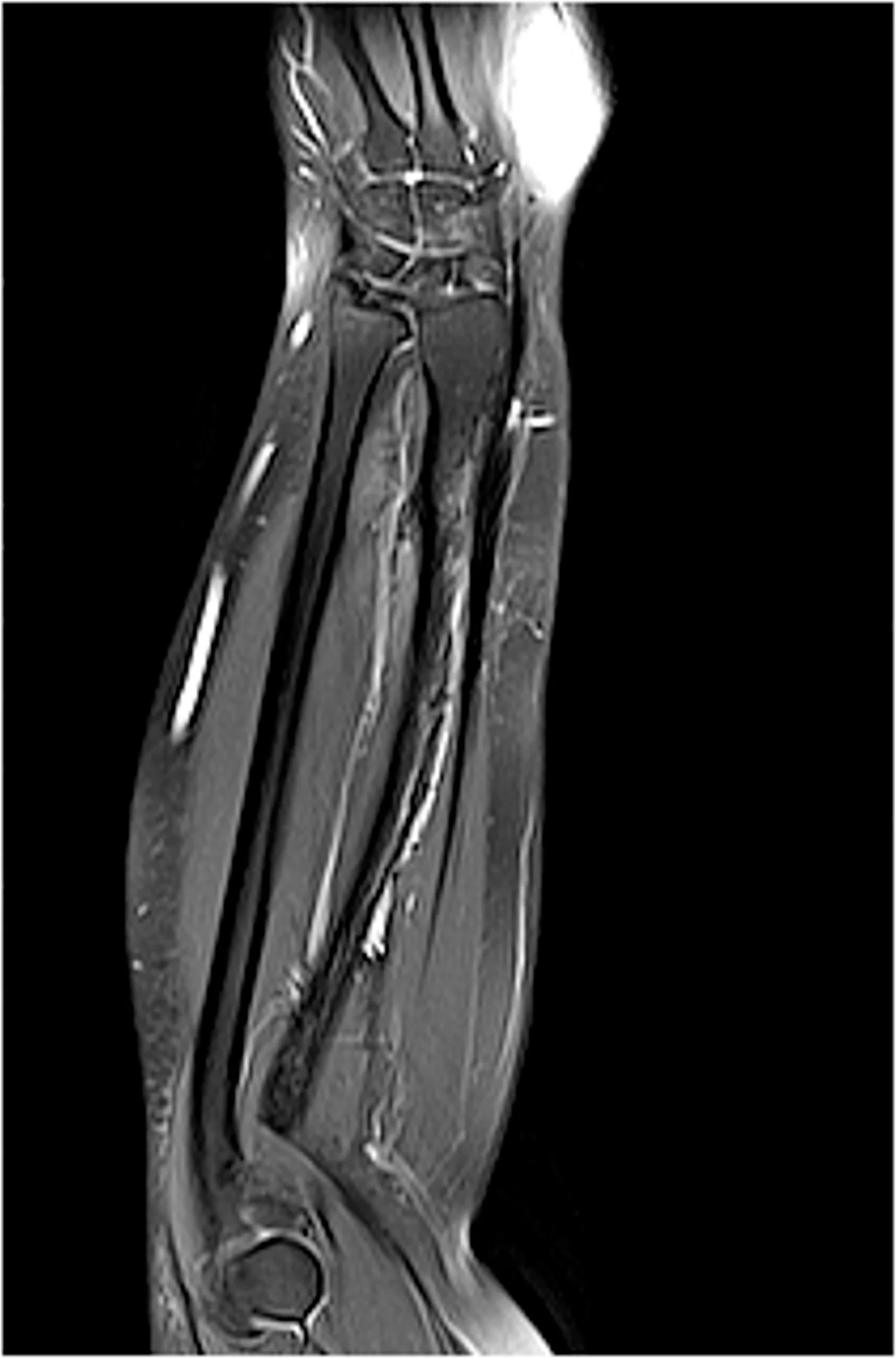
Postoperative MRI of the left forearm demonstrating complete regression of the previously extensive arteriovenous malformation, with no evidence of residual vascular malformation.

**Figure 5:**
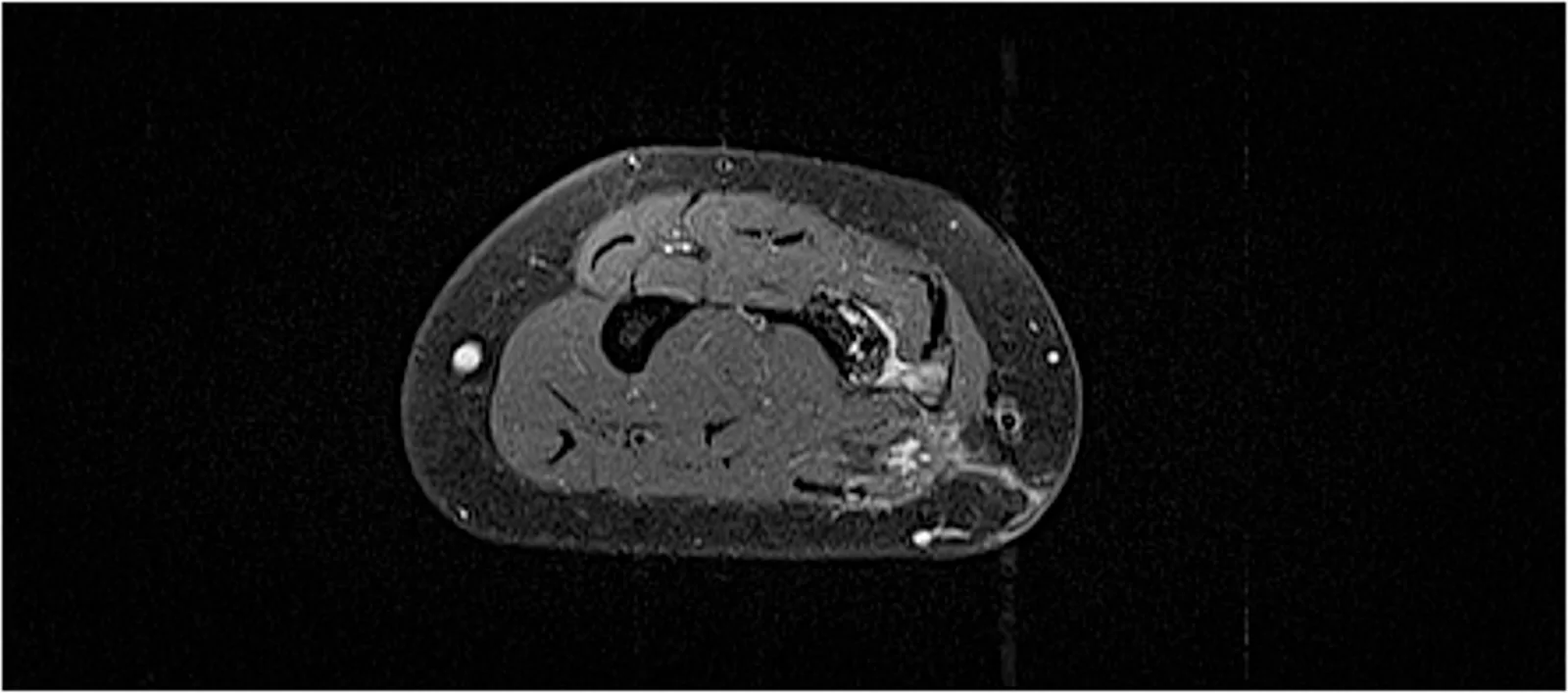
Coronal image showing no evidence of residual vascular malformation.

## Discussion

The first documented case of an intramuscular hemangioma was described by Liston in 1843 in the semimembranosus muscle [5]. Since then, most published reports have consisted of isolated case reports involving various skeletal muscles. By 1983, ~527 cases of intramuscular hemangiomas had been documented in the literature. The lower extremities represent the most frequent site of involvement, (~45%), followed by upper extremities (27%), whereas lesions arising in the head and neck or trunk each account for about 14% [6]. Involvement of the upper limb, particularly around the elbow, remains exceptionally rare [7]. They are typically diagnosed in young adults, with 80%–90% of patients presenting before the age of 30 [8]. Our patient developed the lesion at the age of 34, with recurrence at 44 years of age. Although the exact etiology remains unclear, these lesions are believed to be congenital [9].

Imaging studies play a crucial role in the evaluation of vascular soft tissue lesions and their differentiation from peripheral nerve tumors. Ultrasonography is a useful initial modality because it allows assessment of the lesion and its relationship to adjacent anatomical structures, including nerves, blood vessels, and tendons [10]. However, MRI remains the imaging modality of choice for preoperative assessment because it provides excellent soft tissue contrast and detailed anatomical information, facilitating both diagnosis and surgical planning [11].

Wide excision remains the treatment of choice whenever feasible, as it offers the best chance of cure and minimizes the risk of recurrence. In our case, despite achieving negative surgical margins following the initial excision 10 years ago, the lesion recurred. Given the size of the recurrent lesion, the patient was treated with foam sclerotherapy using 10 ml of 3% polidocanol (Ethoxysclerol), prepared according to the Tessari method, followed by ligation of the radial artery [12].

This case highlights the role of combined treatment with foam sclerotherapy using 3% polidocanol (Ethoxysclerol), prepared according to the Tessari method, followed by radial artery ligation. This combined treatment can achieve an excellent clinical outcome. Complete regression of the lesion was observed 3 months after the surgery, without evidence of residual disease or local recurrence. This report suggests that this combined therapeutic approach may represent an effective treatment option for selected patients with recurrent or extensive lesions when complete surgical excision is difficult or associated with significant morbidity. Larger case series and prospective studies with longer follow-up are needed to establish the long-term efficacy, safety, as well as recurrence rates of this treatment strategy.
